# Effect of a soft wearable robot suit with hip extensor assistance on gait in patients with Parkinson’s disease: a study protocol

**DOI:** 10.3389/fneur.2025.1695612

**Published:** 2025-11-05

**Authors:** Hyun Iee Shin, Ho Seok Lee, Na Young Yun, Haerin Choi, Kyeongmin Lim, Byung Chan Lee, Se Jun Park, Ki Ho Lee, Byeong Jun Cho, Giuk Lee, Don-Kyu Kim

**Affiliations:** 1 Department of Physical Medicine and Rehabilitation, Chung-Ang University Hospital, Seoul, Republic of Korea; 2 Department of Physical Medicine and Rehabilitation, College of Medicine, Chung-Ang University, Seoul, Republic of Korea; 3 School of Mechanical Engineering, Chung-Ang University, Seoul, Republic of Korea; 4 HUROTICS Inc., Seoul, Republic of Korea; 5 Department of Physical Medicine and Rehabilitation, Chung-Ang University Gwangmyeong Hospital, Gwangmyeong-si, Republic of Korea

**Keywords:** Parkinson’s disease, robot assisted gait training, soft wearable robotic suit, gait, hip extensor assistance, rehabilitation

## Abstract

**Background:**

Parkinson’s disease (PD) is a neurodegenerative disorder characterized by progressive motor deficits and gait disturbances. While medication offers symptomatic relief, long-term complications and gradual functional decline remain significant challenges. Robot-assisted training provides intensive, task-specific motor rehabilitation and has shown promise in improving gait for PD patients. Soft wearable robot suits, designed with lightweight, flexible materials, offer enhanced comfort, adaptability, and biomechanical support compared to traditional robots. However, there is limited evidence regarding the effectiveness of hip extensor assistance with soft wearable robots for gait improvement in PD.

**Methods:**

This is a prospective, single-center, single-blind, parallel-group study, and will recruit 34 PD patients. The participants will be assigned to either a robot or control group. Both groups will receive identical rehabilitation interventions, each session comprising 20-min of strength training, 5-min rest, and 20-min of treadmill walking. The rehabilitation program will be applied identically to all participants. The key difference between the groups will be whether participants wear the soft wearable robot suit during treadmill walking session. The intervention will be conducted 2 times per week, a total of 12 sessions for 6 weeks. The H-Medi (HUROTICS, Inc.), a cable-driven soft wearable robot suit will be utilized for the intervention and hip extensor assistance will be applied. For outcome measures, the following assessments will be performed at baseline (T0) and post-intervention (T1): Gait speed, Timed-Up and Go test, Short Physical Performance Battery, Berg Balance Scale, Movement Disorder Society-Unified Parkinson’s Disease Rating Scale, Freezing of Gait Questionnaire, gait parameters, muscle strength and endurance, quadriceps muscle thickness, body composition, cognition, and depression. The primary outcome will be the difference of gait speed from T0 to T1. The secondary outcomes will be the differences of other measures.

**Discussion:**

This study will be the first to assess hip extensor assistance provided by a soft wearable robot suit as a targeted therapy for gait impairment in PD. Results are expected to clarify device usability, safety, and impact on gait. By focusing on hip extension, the findings may help advance personalized gait rehabilitation and inform the design and clinical adoption of future wearable robotic devices for PD.

**Clinical trial registration:**

KCT0010793.

## Introduction

### Background and rationale

Parkinson’s disease (PD) is one of the most common neurodegenerative disorders, marked by the progressive degeneration of dopaminergic neurons in the substantia nigra and the resulting dopamine deficiency (1). PD is clinically characterized by the presence of bradykinesia in combination with either resting tremor, rigidity, or both, and is frequently accompanied by non-motor symptoms such as cognitive decline, depression, sleep disturbances, and autonomic dysfunction (2). Reduced motor function caused by dopaminergic deficits leads to impaired motor automaticity, which ultimately results in gait disturbances and adversely affects the quality of life in patients with PD (3). Although dopaminergic pharmacotherapy remains the gold standard of treatment, it offers only symptomatic relief and is associated with long-term complications such as levodopa-induced dyskinesia. Surgical interventions, including deep brain stimulation, provide benefit for selected individuals, yet most patients experience gradual functional deterioration despite therapy. The global prevalence of PD is rising significantly, mainly driven by population aging, increased disease duration (4–6). Therefore, there is a need for disease-modifying treatments and integrated rehabilitative strategies to preserve motor function, including gait, and to improve quality of life in patients with PD.

Robot-assisted training has recently emerged as a promising rehabilitation technology designed to deliver intensive, repetitive, and task-specific motor training to improve motor function, mobility, and gait restoration (7). One of the well-established pathophysiological goals of utilizing robot-assisted training to improve motor function is to promote neuroplasticity; therefore, it is widely used for diseases affecting the central nervous system, such as PD or stroke (7, 8). Robotic devices can assist or resist movements as needed, ensure consistent training intensity and dosage, and provide real-time feedback, thereby enhancing physical performance and motivation compared to conventional rehabilitation conducted by physical therapists (9). These benefits may arise from the ability to deliver high-repetition, task-specific, and precisely controlled interventions, reduce therapist burden, support patient engagement through individualized programming, and provide reliable, objective monitoring features that conventional therapy may lack due to variability in manual guidance and therapist workload. Traditionally, robots are primarily categorized into two types, exoskeletal and end-effector robots. However, several limitations restrict their broader adoption and clinical impact. Exoskeletal robots are often characterized by their weight, mechanical complexity, and high cost, which can make achieving a comfortable and precise fit challenging across diverse users (10). Misalignment between the axes of robotic joints and the wearer’s anatomical joints can induce unintended forces and torques, leading to discomfort, potential safety hazards, and even long-term injury with frequent use. Moreover, these devices generally lack portability due to their bulk and design complexity, restricting their practical application primarily to controlled, specialized clinical or research environments (11). End-effector robots, while generally simpler and safer regarding alignment, are limited in their ability to isolate control of individual joints and may lead to less natural movement patterns, offering insufficient support for patients with severe motor deficits (12).

To address the limitations of exoskeletal and end-effector robots, there is a growing need for the development and implementation of soft wearable robots in clinical rehabilitation. Soft wearable robots are designed using flexible, lightweight materials that conform closely and naturally to the human body, thereby addressing key limitations of traditional rigid robotic systems, including excessive weight, mechanical complexity, poor fit, and limited portability (13). By reducing overall weight and mechanical complexity, these devices may provide a more natural and comfortable interface between the user and the device, enabling flexible and personalized assistance. In addition, soft wearable robots utilize flexible, lightweight materials and cable-driven actuation systems, such as Bowden cables, to transmit mechanical assistance efficiently while allowing free and natural joint motion. This design minimizes interference with voluntary movement, which is particularly critical for patients with PD, who often experience impaired motor automaticity and fluctuations in gait initiation and rhythm, including freezing of gait (FoG). FoG, characterized by a temporary inability to step forward despite the intention to walk, is a major contributor to falls, imbalance and reduced mobility in PD (14). It is well known that external cues can help overcome impaired gait function by providing additional sensory input that facilitates the activation of locomotor networks and restores step rhythm (15, 16). In this context, soft wearable robots can serve not only as physical assistive devices but also as sources of external cueing through synchronized mechanical assistance and sensory feedback. Equipped with sensors such as inertial measurement units (IMU), these robots can detect gait phases in real time and deliver timely hip assistance aligned with the user’s walking cycle (17, 18). Through the combined effects of mechanical aid and external cueing, soft wearable robots have the potential to promote smoother, more stable, and energy-efficient gait patterns in patients with PD.

Recently, robot-assisted training has become increasingly utilized for patients with PD to address motor impairments, including gait dysfunction. Current applications of robot-assisted training in patients with PD have focused on improving key gait parameters (9, 19). Picelli et al. have reported robot-assisted gait training showed significant effects on 10-meter walk test, stride length, cadence, balance, and Movement Disorder Society-Unified Parkinson’s Disease Rating Scale (MDS-UPDRS) Part III (20–23). Sale et al. and Furnari et al. also showed similar results (24, 25). A recent systematic review and meta-analysis also highlighted the benefits of robot-assisted training in improving outcomes such as the Berg Balance Scale (BBS), Activities-specific Balance Confidence Scale, 10-meter walk test, gait speed, stride length, cadence, and MDS-UPDRS Part III. However, the overall level of evidence was low. Additionally, the Timed Up and Go test (TUG) and the 6-min walk test were assessed, with findings indicating a very low level of evidence (9).

Additionally, studies employing soft wearable robots are increasingly being reported. Previous research on soft wearable robotic suits for gait assistance has primarily focused on providing hip flexor or extensor support to improve mobility in diverse populations, including healthy older adults and patients undergoing rehabilitation after neurological conditions These studies have demonstrated that such devices can reduce muscular effort, enhance joint kinematics, and improve gait efficiency and symmetry by augmenting hip flexion and/or extension during walking (26–28). Despite these promising results, the application of soft wearable robotic suits specifically targeting hip flexor or extensor assistance in patients with PD remains limited. Kim et al. described the potential of soft wearable robotic apparel that provides hip flexion assistance to mitigate FoG in an individual with PD (29). A randomized control study conducted by Kawashima et al. also utilized a wearable robot that assists hip flexion and extension to improve gait function. Although the robot group showed improvements in distance and gait speed during the 3-min walk test, these changes were not statistically significant compared with the control group (30). PD presents distinct gait impairments, including reduced hip extension during the stance phase, which contributes to decreased stride length and mobility limitations (31). Unlike previous study that employed robotic devices assisting both hip flexion and extension, this study will focus solely on hip extensor assistance. This targeted approach is based on the biomechanical understanding that enhancing hip extension can more effectively improve propulsion during walking. We hypothesize that by specifically augmenting the impaired hip extensor function characteristic of PD gait, the robotic device will enhance hip extension, thereby promoting greater propulsion, increased stride length, improved stability, and overall enhancement of gait mechanics (32, 33). In addition, we suggest that these improvements in gait could be further facilitated and consolidated through neuroplasticity (7, 34). Ultimately, this focused assistance may lead to enhanced mobility and more efficient walking in patients with PD.

Therefore, this study aimed to evaluate the impact of a soft wearable robotic suit with hip extensor assistance on gait in patients with PD.

## Methods

### Trial design

This is a prospective, single-center, single-blind, parallel-group trial. Participants will be assigned to two groups, in a 1:1 ratio: the robot group and the control group. The intervention session times will be pre-assigned as either robot or control sessions, with an equal number of slots for each group. Participants will then select their preferred session time according to their personal schedules, without knowing which sessions are designated as robot or control. Their group allocation will be determined based on this selection. As randomization is not employed and group assignment is based on self-selection, potential selection bias and confounding may occur. However, since session times for both groups are evenly distributed and baseline demographic and clinical characteristics of participants will be collected, we suggest that potential bias can be minimized. In the analysis of study results, we will apply appropriate statistical adjustments for any baseline differences. One research investigator will manage the session schedule and contact participants. This investigator, physical therapists conducting the intervention, and the participants will not be blinded to group assignments. All other investigators including outcome assessors, investigators involved in data interpretation and analyses, and the principal investigator (PI) will remain blinded throughout the study. Group assignments will be unblinded only after completion of the final statistical analyses, or during the study if a serious adverse event occurs, with the approval of the PI.

### Study setting

This study will be conducted at Chung-Ang University Hospital in Seoul, Republic of Korea, in accordance with the principles of Good Clinical Practice and the Declaration of Helsinki.

### Eligibility criteria

The inclusion criteria are as follows:

1 Patients clinically diagnosed with idiopathic PD according to the UK Parkinson’s Disease Society Brain Bank Diagnostic Criteria (35).
2 Patients classified as modified Hoehn and Yahr Stage 2–3.
3 Patients who have provided informed consent and voluntarily signed the written consent form to participate in the study.

The exclusion criteria are as follows:

1 Patients with cognitive impairment [Mini-Mental State Examination (MMSE) score <24].
2 Patients with musculoskeletal disorders that impair independent walking.
3 Patients with limb amputation.
4 Patients with medical conditions that affect the performance of activities of daily living.
5 Patients with vestibular disorders or a history of vertigo.
6 Patients deemed inappropriate for participation in the study by the investigator.

### Interventions

The participants will receive a 45-min rehabilitation exercise intervention session consisting of 20-min of strength training, a 5-min rest period, and 20-min of treadmill walking. Strength training will be conducted in a one-on-one setting with a skilled physical therapist and included hamstring and quadriceps stretching, one-leg bridging, hip abductor strengthening, quadriceps strengthening, bird-dog exercises, squatting, and one-leg standing exercises. During the treadmill walking session, participants were secured with a harness to ensure safety and walked at an individually self-selected speed within target of a moderate intensity range (Borg Rating of Perceived Exertion scale, RPE 11–13). The rehabilitation exercise program will be applied identically to all participants. The only difference between the two groups will be whether participants wear the soft wearable robot suit during the 20-min treadmill walking session. The intervention will be conducted 2 times per week, a total of 12 sessions for 6 weeks. We will review each participant’s medication schedule to determine their PD medication on/off periods. The intervention will be conducted during the “on” periods for each participant.

**Figure 1:**
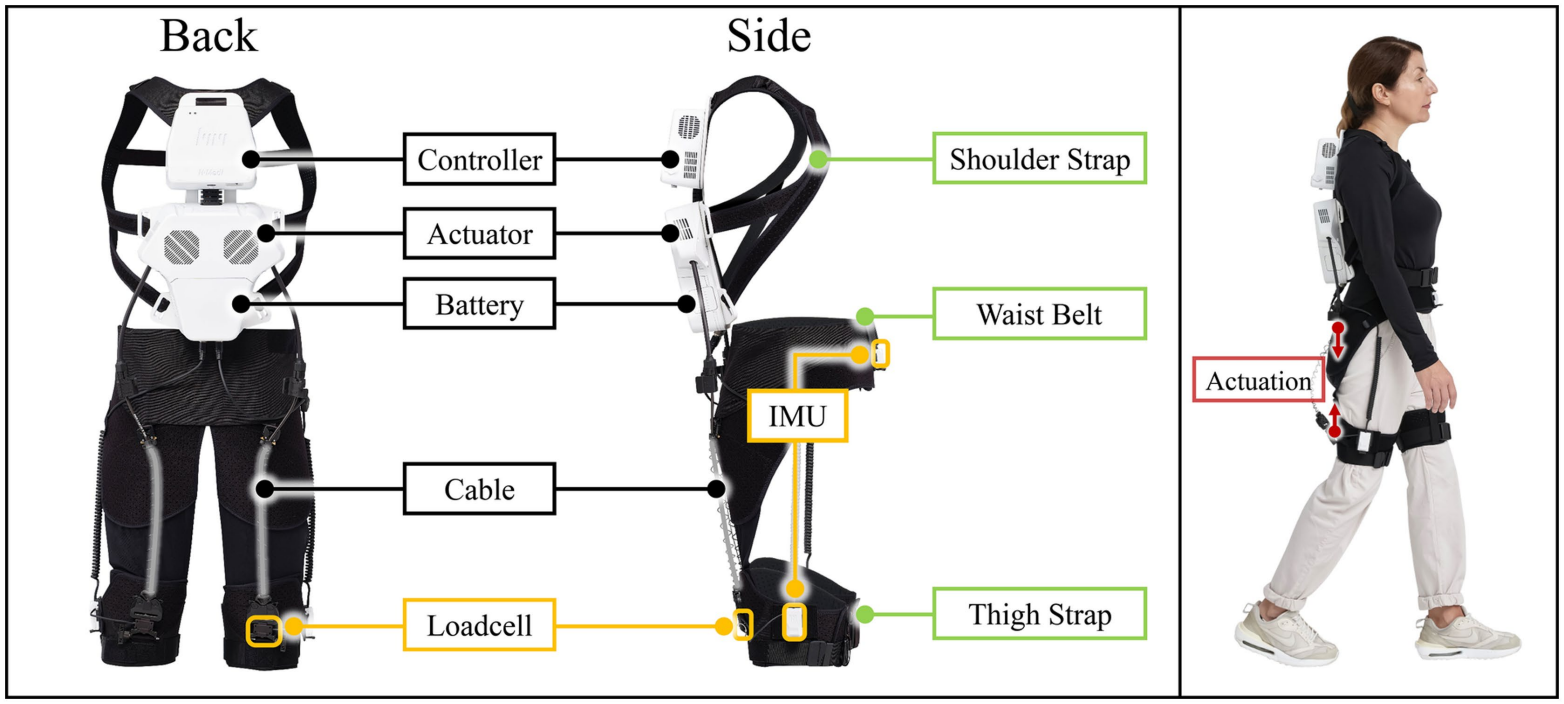
The cable-driven soft wearable robot suit (H-Medi), showing key components such as including main unit (black boxes), sensors (yellow boxes), straps (green boxes), and directions of applied forces (red boxes).

The intervention utilized the H-Medi (HUROTICS, Inc.), a cable-driven soft wearable robot suit designed for gait assistance (Figure 1). The system consists of a main module housing two motors and a battery, connected to a waist belt and a pair of thigh straps, with a total weight of 4.5 kg. Multiple apparel sizes were available to ensure a proper fit for each participant. Three IMU sensors, placed on the abdomen and both thighs, monitored the wearer’s movements in real time. The assistive force is generated by motors in the back-worn main module, a placement that positions the actuators near the body’s center of mass and minimize inertial burden. This force is transmitted to the thighs via a flexible Bowden cable system. The waist belt serves as the proximal anchor, while the distal end of the cable connects to an attachment point on the thigh strap. This provides targeted assistance, enabling the force to be precisely applied to desired muscle groups without interfering with the user’s natural movements. In this study, the H-Medi was configured to assist hip extension with the goal of enhancing forward propulsion and gait stability, thereby increasing gait speed in patients with PD. Although the device can deliver a maximum assistive force of 200 N (≈ 30 Nm), the experimental range was set between 30 N (≈ 4.5 Nm) and 130 N (≈ 19.5 Nm). Force magnitude was adjusted by the physical therapist based on clinical judgment and was progressively increased from 30 N throughout training. This clinical judgment was guided by a clinical judgment integrating the participant’s subjective feedback on exertion (Borg RPE scale), the therapist’s observation of gait stability without excessive compensatory movements, and a qualitative assessment of overall movement quality (e.g., observed changes in stride length, stability, and posture). Treadmill speed was concurrently raised to augment the rehabilitation effect, with both parameters modulated according to each participant’s self-selected walking speed and Borg RPE scale. The assistive force profile followed a half-sine waveform defined by three key gait timings from prior optimization studies in healthy adults: onset at approximately 13% of the gait cycle before heel strike, peak at 21% after heel strike, and offset at 38% after heel strike (36). The gait cycle was estimated based on hip-centric events, such as Maximum Hip Extension (MHE) and Maximum Hip Flexion (MHF), detected by the IMU sensors on the abdomen and thighs. This profile was further individualized based on participant feedback and the therapist’s expertise to ensure optimal assistance.

The criteria for discontinuing the intervention are as follows:

1 Voluntary withdrawal by the participant.
2 Missing a total of 3 out of 12 intervention sessions.
3 Consecutively missing 2 intervention sessions.
4 Discontinuation due to significant adverse events.
5 Research investigator’s judgment that the continuation of the intervention is unsuitable.

The rehabilitation exercise session has been established as effective for patients with PD, regardless of the use of the soft wearable robot. Therefore, participants will be informed that assignment to either study group may not adversely affect their physical impairments or functional levels during the intervention period. Additionally, since all interventions will take place within the hospital, research coordinators will maintain periodic contact with participants via phone calls to remind them of upcoming interventions.

Participants will maintain their usual dosage of medication for their underlying conditions, including PD. Beyond the exercise intervention implemented in this study, any additional physical activities conducted at medical centers, including rehabilitation centers, are prohibited.

### Outcomes

Baseline evaluations (T0) will be conducted within 7 days before the initiation of the intervention. Post-intervention evaluations (T1) will be conducted within 7 days after the final intervention session.

The primary outcome will be the change in gait speed from T0 to T1. Gait speed will be measured as the average value from two trials of the 10-meter walk test. This test is sensitive to mobility impairment, predicts disability and falls, and is feasible for use in clinical settings, demonstrating both reliability and validity (37, 38).

The secondary outcomes will be the change from T0 to T1 of the following assessments: 4-meter walk test, TUG, Short Physical Performance Battery (SPPB), BBS, MDS-UPDRS, Freezing of Gait Questionnaire (FoGQ), gait parameters measured by an IMU sensor-based gait analysis system (Human Track, R. Biotech Co. Ltd., Seoul, Korea) (39) and the GAITRITE system (CIR Systems, PA, United States) (40), muscle strength and endurance measured by isokinetic dynamometer Biodex system (Biodex System 4, Biodex Medical Systems, Shirley, NY, United States) (41), thickness of quadriceps muscles measured by ultrasound, body composition measured by Dual-energy X-ray absorptiometry (DEXA) (42), cognitive function measured by Mini-Mental State Examination (MMSE), and depression measured by Beck Depression Inventory (BDI). In addition, questionnaires assessing comfort and usability, along with participants’ feedback, will be collected after the completion of the intervention sessions.

The TUG evaluates gait, balance, and fall risk. Participants will rise from a chair, walk 3 meters to a cone, turn, return to the chair, and sit down. Completion time (seconds) will be recorded (43). The SPPB will assess balance, walking ability, and transfers. Each component is scored on a 0–4 scale, with a total score ranging from 0 to 12, where higher scores indicate better performance. The BBS assesses functional standing balance through 14 tasks of increasing difficulty, including sitting, standing, single-leg stance, and positional changes. Each item is scored on a 0–4 scale, with a maximum total score of 56, where higher scores indicate better balance ability (44).

The MDS-UPDRS is a comprehensive tool for evaluating Parkinson’s disease across four domains: (I) non-motor experiences of daily living, (II) motor experiences of daily living, (III) motor examination, and (IV) motor complications. Parts I and II are based on patient or caregiver questionnaires, while Parts III and IV are assessed by a clinician. Each item is rated on a 0–4 scale, with higher scores indicating greater impairment, allowing for a detailed assessment of both motor and non-motor symptoms (45). The FoGQ is a brief, reliable self-reported instrument designed to assess FoG in individuals with Parkinson’s disease. It consists of six items evaluating the frequency, duration, and impact of FoG episodes, such as start hesitation and turning hesitation. Each item is scored on a 0–4 scale, with higher scores indicating more severe symptoms (46).

The gait parameters measured by the IMU sensor-based gait analysis system and the GAIRRite system will include stride length, step length, cadence, proportion of single and double support, proportion of swing and stance phases, and the range of motion (ROM) of hip, knee, and ankle joint angles. The parameters measured by the Biodex system will include the number of repetitions, peak torque (N·m), peak torque/body weight (%), maximal repetition total work (J), coefficient of variation (CV, %), average power (W), and total work (J). Measurement will be obtained for both knee flexion and extension at angular velocities of 60°/second and 180°/second.

Quadriceps muscle thickness will be measured by ultrasound. A single trained investigator will perform all measurements. The probe will be placed transversely, 10 cm above the superior border of the patella, along the line from the anterior superior iliac spine to the patella (47, 48). Body composition will be assessed using DEXA, including measurements of body mass index (BMI), skeletal muscle mass index (SMI), total body fat percentage, fat mass index, and bone mineral density (BMD).

Cognitive function and mood are often impaired patients with PD (49). Additionally, exercise may have a positive effect on these dysfunctions (50–52). Therefore, we included cognitive and depression as secondary outcomes. Cognitive function will be assessed using the MMSE, a widely used screening tool for global cognitive status that is sensitive to cognitive decline in patients with Parkinson’s disease (53). Depression will be evaluated using the BDI, a validated self-report questionnaire that reliably measures the severity of depressive symptoms in this population (54). Both tools have been extensively used in PD research and are practical for clinical and research settings.

### Participant timeline

**Figure 2:**
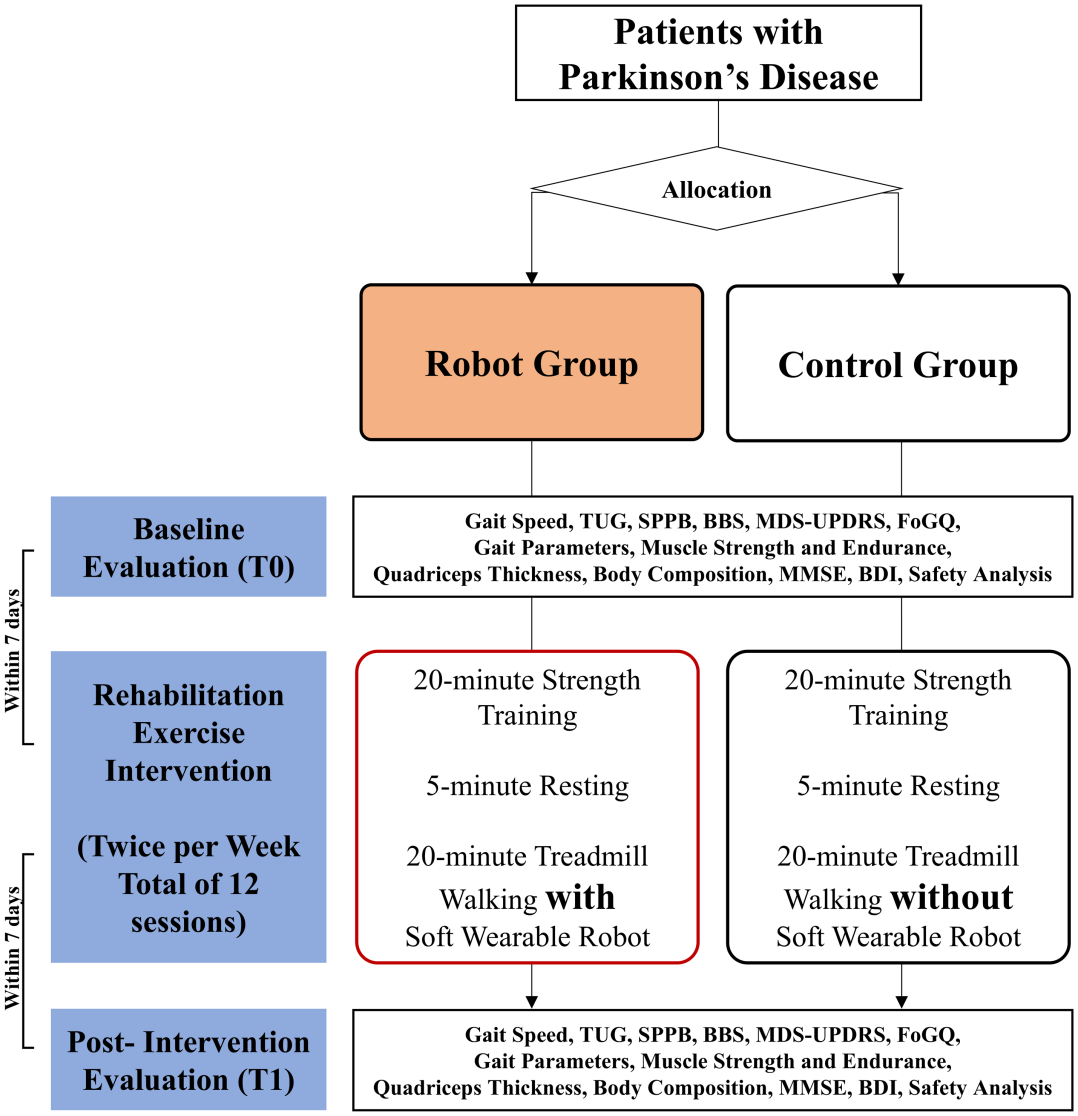
Design and flowchart of the study.

Figure 2 shows the brief design and flowchart of the study. All participants will be enrolled in the study after completing the predefined screening process. Following enrollment, baseline evaluations will be conducted. Within 7 days of these evaluations, the rehabilitation exercise intervention will begin, consisting of 12 sessions over 6 weeks. After the intervention, post-intervention evaluations will be conducted within 7 days. The specific timeline of participants is shown in Table 1.

**Table 1:** Timeline of enrollment, interventions, and assessments of the study.

Time point	Enrollment	Baseline (T0)	Intervention	Post-intervention (T2)
Informed consent	O			
Eligibility screen	O			
Group assignment		O		
Assessments				
Gait speed		O		O
TUG		O		O
SPPB		O		O
BBS		O		O
MDS-UPDRS		O		O
FoGQ		O		O
Gait parameters		O		O
Muscle strength and endurance		O		O
Quadriceps thickness		O		O
Body composition		O		O
MMSE		O		O
BDI		O		O
Safety analysis		O		O
Application of intervention				
Intervention			O	

TUG, Timed-Up and Go test; SPPB, Short Physical Performance Battery; BBS, Berg Balance Scale; MDS-UPDRS, Movement Disorder Society-Unified Parkinson’s Disease Rating Scale; FoGQ, Freezing of Gait Questionnaire; MMSE, Mini-Mental State Examination; BDI, Beck Depression Inventory.

### Sample size

The primary outcome of this study is the change in gait speed between T0 and T1, which has been widely used in previous studies (9). The power of the study (1–*β*) was set at 80%, with a significance level (*α*) of 5%. The required sample size was calculated using Lehr’s formula (55). The clinically significant effect size (*δ*) was been designated as 0.18, with an expected standard deviation (*σ*) of 0.17 (22, 38). Considering an anticipated follow-up rate of 80%, the final calculated sample size was 17 participants per each group, totaling 34 participants.

### Recruitment

Study participants will be recruited through:

1 Notices posted on the Chung-Ang University Hospital bulletin boards
2 Department of Physical and Rehabilitation Medicine outpatient clinic of the Chung-Ang University Hospital
3 Notices posted on the bulletin boards of the Korean Parkinson’s Disease Association.

Eligibility to participate in the study will be determined by the research investigators based on the predefined criteria. Participants will be informed about the current treatment guidelines for mobility function in patients with Parkinson’s disease, as well as the potential benefits and risks associated with this study.

### Statistical methods

Demographic and clinical characteristics will be summarized using frequencies and percentages for categorical variables and means with standard deviations (SD) for continuous variables. The Shapiro–Wilk test will be used to assess the normality of continuous variables. To compare baseline characteristics between the intervention and control groups, an independent t-test will be applied for normally distributed variables, while the Mann–Whitney U test will be used for non-normally distributed variables.

All participants who undergo the intervention will be included in the intention-to-treat (ITT) population, which will also serve as the basis for safety analyses. Participants in the ITT population who complete both baseline and post-intervention assessments will comprise the full analysis set (FAS). Efficacy analyses will be conducted based on the FAS.

For efficacy analyses, the normality of outcome variables will be assessed using the Shapiro–Wilk test. Baseline characteristics will be included as covariates for adjustment in the statistical models. Additionally, comparisons between the robot (intervention) and control groups under each condition will be performed using either the independent t-test or the Mann–Whitney U test, depending on the distribution of the data. To compare post-intervention outcomes between the groups while adjusting for baseline values, analysis of covariance (ANCOVA) will be used. The post-intervention measurement will be the dependent variable, group assignment the independent factor, and the baseline measurement of the outcome will be included as a covariate. This approach accounts for any baseline imbalances and increases statistical power. Assumptions of normality and homogeneity of regression slopes will be assessed prior to analysis. Statistical significance will be set at a two-sided *p*-value of <0.05 for all analyses.

### Data collection, management, and monitoring

To ensure high data quality, all research investigators and assessors held a series of meetings to discuss and establish standardized assessment protocols. Subsequently, comprehensive training sessions were conducted to ensure that all assessors were thoroughly familiar with the standardized methodologies. Furthermore, we carefully document and distribute standardized research evaluation protocols to facilitate the continuous review of evaluation methodologies and data collection instruments.

All study data will be collected using a standardized electronic Case Report Form (eCRF), and participants will be identified solely by a study-specific serial number. Personal information and collected data will be kept strictly confidential under the supervision of the research PI. Data will be stored in password-protected files within a secure, locked facility. An independent researcher, not involved in the main study activities, will regularly monitor the data. The data management team will develop a data validation plan and will be responsible for query management, coding of adverse events, and reconciliation of serious adverse events.

Regularly scheduled plans for auditing trials are not in place. However, audits may be conducted at any time by an internal auditing organization within the Institutional Review Board (IRB) of Chung-Ang University Hospital, where the clinical trial is being conducted. The audit process will be independent of the investigators and sponsor. Data monitoring committee, consisting of the PI and monitoring agents from the participating hospital, conducts data monitoring every 3 months, with additional irregular monitoring in the event of serious adverse events. The sponsor has played no role in the study’s design and not involved in the collection, analysis, interpretation of data, or writing of the manuscripts.

### Adverse events and harms

Adverse events during this study may include musculoskeletal discomfort, dizziness, fatigue, or general symptoms that could be reported in patients with PD. The most serious expected adverse event is falling. During the exercise intervention, strength training will be conducted in a one-on-one setting with a physical therapist who will continuously monitor for falls. During treadmill training, a safety harness will be used to prevent falls. All adverse events will be monitored. All adverse events that occurred will be reported to PI and ethics committee of the participating hospital within 7 days.

### Ethics and dissemination

Prior to inclusion of participants, we obtained IRB approval for this study (IRB number: 2401–020-587). Informed consent will be obtained from all participants by research investigators prior to their inclusion in the study. The contents of the informed consent form, which were approved by the IRB, include an explanation of the study’s purpose, potential benefits and risks, and provide clear contact information for both the investigator and the IRB in case participants have any questions during the study.

This trial has been registered at Clinical Research Information Service (CRIS) in South Korea (KCT0010793). Any important modifications to the protocol will be promptly communicated to all relevant parties, including research investigators, the ethics committee of the participating hospital, and trial registration, under the responsibility of the PI. Participants will also be informed if the changes directly affect their involvement. The results of this study are expected to be published within 1 year of its completion. The PI and the researcher designated for statistical analysis will have access to the final dataset. Researcher affiliated with HUROTICS Inc. will be prohibited from participating in data analysis. Any data required to support the findings of this study will be available from the corresponding author upon reasonable request.

## Discussion

This study will investigate the effect of a soft wearable robot suit with hip extensor assistance on gait in patients with PD. To the best of our knowledge, this will be the first study to specifically evaluate the impact of hip extensor assistance delivered through a soft wearable robot suit in this population.

We anticipate several advantages that distinguish this study from previous research. First, it will provide evidence on the overall impact of a soft wearable robot suit in patients with PD, including its comfort, usability, safety, and effectiveness in real-world gait training. Second, by focusing specifically on hip extensor assistance, the study will directly address gait deficits characteristic of PD and allow us to clarify its effects on gait parameters such as propulsion, stride length, gait symmetry, and gait speed. By integrating findings from prior studies, the results of this study may also help identify the potential unique benefits of hip extensor assistance compared with other modes, such as flexor-only or combined flexor–extensor assistance. Furthermore, the outcomes of this study may contribute to the development of personalized strategies for promoting gait function in PD patients using wearable robotic devices. Understanding how targeted assistance to a specific joint movement affects gait mechanics will help optimize the design and control algorithms of future devices. Lastly, our findings could provide valuable guidance for future clinical trials and research employing wearable robot suits in gait rehabilitation, ultimately informing evidence-based recommendations for their clinical adoption in PD.
